# Two Separate Brain Networks for Predicting Trainability and Tracking Training-Related Plasticity in Working Dogs

**DOI:** 10.3390/ani14071082

**Published:** 2024-04-02

**Authors:** Gopikrishna Deshpande, Sinan Zhao, Paul Waggoner, Ronald Beyers, Edward Morrison, Nguyen Huynh, Vitaly Vodyanoy, Thomas S. Denney, Jeffrey S. Katz

**Affiliations:** 1 Auburn University Neuroimaging Center, Department of Electrical & Computer Engineering, Auburn University, Auburn, AL 36849, USA; szz0011@tigermail.auburn.edu (S.Z.); rjb0018@auburn.edu (R.B.); nph0013@auburn.edu (N.H.); dennets@auburn.edu (T.S.D.J.);; 2 Department of Psychological Sciences, Auburn University, Auburn, AL 36849, USA; 3 Alabama Advanced Imaging Consortium, Birmingham, AL 36849, USA; 4 Center for Neuroscience, Auburn University, Auburn, AL 36849, USA; 5 Department of Psychiatry, National Institute of Mental Health and Neurosciences, Bangalore 560029, India; 6 Department of Heritage Science and Technology, Indian Institute of Technology, Hyderabad 502285, India; 7 Canine Performance Sciences Program, College of Veterinary Medicine, Auburn University, Auburn, AL 36849, USA; waggolp@auburn.edu; 8 Department of Anatomy, Physiology & Pharmacology, Auburn University, Auburn, AL 36849, USA; morriee@auburn.edu (E.M.); vitalyvodyanoy@auburn.edu (V.V.)

**Keywords:** canine, dog, resting state, functional MRI, functional connectivity, comparative biology

## Abstract

**Simple Summary:**

The expense associated with training detection and service dogs is significant. By employing resting-state functional resonance imaging technique, a non-invasive method capable of probing brain function, we can identify the critical brain regions linked to selecting dogs inclined towards successful training. These biomarkers identified before commencement of training predict successful trainability and hence reduce training costs by obviating the need to invest in dogs that are unlikely to be successful. Furthermore, our research extends to elucidating the identified brain regions in dogs that exhibit homologous functions to those found in the human brain, offering valuable insights into the evolutionary parallels between humans and our closest animal companions.

**Abstract:**

Functional brain connectivity based on resting-state functional magnetic resonance imaging (fMRI) has been shown to be correlated with human personality and behavior. In this study, we sought to know whether capabilities and traits in dogs can be predicted from their resting-state connectivity, as in humans. We trained awake dogs to keep their head still inside a 3T MRI scanner while resting-state fMRI data was acquired. Canine behavior was characterized by an integrated behavioral score capturing their hunting, retrieving, and environmental soundness. Functional scans and behavioral measures were acquired at three different time points across detector dog training. The first time point (TP1) was prior to the dogs entering formal working detector dog training. The second time point (TP2) was soon after formal detector dog training. The third time point (TP3) was three months’ post detector dog training while the dogs were engaged in a program of maintenance training for detection work. We hypothesized that the correlation between resting-state FC in the dog brain and behavior measures would significantly change during their detection training process (from TP1 to TP2) and would maintain for the subsequent several months of detection work (from TP2 to TP3). To further study the resting-state FC features that can predict the success of training, dogs at TP1 were divided into a successful group and a non-successful group. We observed a core brain network which showed relatively stable (with respect to time) patterns of interaction that were significantly stronger in successful detector dogs compared to failures and whose connectivity strength at the first time point predicted whether a given dog was eventually successful in becoming a detector dog. A second ontologically based flexible peripheral network was observed whose changes in connectivity strength with detection training tracked corresponding changes in behavior over the training program. Comparing dog and human brains, the functional connectivity between the brain stem and the frontal cortex in dogs corresponded to that between the locus coeruleus and left middle frontal gyrus in humans, suggestive of a shared mechanism for learning and retrieval of odors. Overall, the findings point toward the influence of phylogeny and ontogeny in dogs producing two dissociable functional neural networks.

## 1. Introduction

For over tens of thousands of years, dogs (*Canis familiaris*) have played versatile roles in human societies, from companionship to performing specific tasks such as detection and therapy work, underscoring their unique trainability and social intelligence [1]. For example, sniffer dogs have been trained to detect explosives, as hearing dogs for alerting people who are deaf to important sounds [2], as therapy dogs for supporting language-impaired children [3] and those with stress- and anxiety-related disorders [4], etc. Consequently, the ease with which dogs can be trained to perform various tasks, as well as their general behavioral capabilities such as hunting and retrieving, become critical parameters for selecting dogs for training. Despite the importance of canine capabilities to human society and the capital costs incurred in training dogs [5,6], research into the neural basis for the behavior of dogs and their trainability is sparse. Such a research endeavor is important for the following reasons. First, it could potentially lead to procedures involving non-invasive measurement of canine neural function as a criterion for selecting dogs to be trained, thereby limiting capital expenditure on less trainable dogs or dogs with less favorable behavioral capabilities. Training a service dog that is effective in its duties can be quite costly, with many experts estimating that the expenses could reach figures from $20,000 to $50,000 [7]. Therefore, by pinpointing suitable candidates for detection work early, the average cost of training could be significantly reduced. Second, a scientific account of the neural structures/processes supporting behavior in dogs could be evaluated with respect to similar literature in humans and other species for understanding the evolutionary role of brain–behavior relationships. This comparative evaluation is particularly relevant for dogs since they are a unique species because they have socially co-evolved with humans for thousands of years [8].

One of the most widely used non-invasive tools for investigating the neural basis of behavior in humans is functional magnetic resonance imaging (fMRI), which is based on the principle that changes in the local concentration of the paramagnetic deoxygenated hemoglobin due to neural activity leads to enhancement in the magnetic resonance signal. While functional magnetic resonance imaging (fMRI) has revolutionized our understanding of the neural basis of behavior in humans, its application to studying the canine brain, particularly through non-invasive resting-state fMRI (RS-fMRI), represents a novel frontier in comparative neurobiology. Of particular interest to brain–behavioral relationships is the fMRI signal in the absence of a goal-directed task, known as resting-state fMRI (RS-fMRI), which displays spatially correlated structure-forming distributed brain networks [9]. In humans, the functional connectivity (FC) between brain regions in such networks (measured through temporal correlation between fMRI time series from those regions), has been shown to co-vary with various behavioral variables (e.g., cognitive abilities, attention, working memory, cognitive control) [10,11,12,13,14]. Szabó et al., 2019 [15] and Beckmann et al., 2020 [16] employed resting-state fMRI and the independent component analysis (ICA) method to demonstrate the presence of resting-state networks (RSNs) in dogs, both in the awake and anesthetized state, like those found in humans and other animals. While those studies have been conducted, they have not yet shown the functional properties or interactions between those networks or any correlation with behavioral data. In our work, we extended this concept from humans to dogs, and surmised that fMRI-based resting-state functional connectivity in brain networks obtained from the dog brain would correlate with canine behavior.

The methodological challenges involved in measuring fMRI-based functional connectivity in the dog brain are quite daunting. First, head movement poses a big problem for fMRI since the displacement of the head from one acquisition to the next, if not corrected for, can appear like a change in image intensity which is unrelated to underlying neural activity. Therefore, fMRI studies employing animals have either immobilized them [17,18] or anesthetized them. The former reduces the comparative validity of the experiment (e.g., humans are not immobilized) and may make the experiment less ethologically valid, while the latter has been proved to alter neural activity and connectivity [19,20]. Therefore, imaging awake dogs is the best workable option. Recent studies have made strides in this regard. Both our group [8,21,22,23] and three other groups [7,24,25,26,27,28,29,30,31,32,33,34,35] have been successful in training dogs to keep their head still inside the MRI scanner while fMRI data is acquired. Specifically, we showed the existence of resting-state brain networks in dogs by scanning them in a fully conscious and unrestrained state. Critically, we employed optical head motion tracking with an external camera device to record and account for head motion, which is inevitable even if the dog is trained to keep its head still. Using this paradigm enabled us to non-invasively measure whole brain functional connectivity in awake dogs.

The objectives of the current study were twofold. First, we wanted to discover the resting-state brain networks whose change in the strength of connectivity during a canine training regimen mirrored corresponding changes in their behavior. Second, we were interested in investigating whether resting-state brain networks estimated from fMRI data acquired before the commencement of the training regimen were able to predict whether a given dog would eventually graduate to become a detector dog or not. In order to achieve these objectives, we designed a longitudinal experimental paradigm where fMRI data and behavioral assessments were acquired at multiple time points (TPs) across the time of participation of dogs in this study. The first time point (TP1) was prior to the dogs entering formal working detector dog training, but after 1–3 months of MRI training [21,22] to keep their head still inside the scanner. The second time point (TP2) was soon after formal detector dog training which lasted about 3 months. The third time point (TP3) was three months post detector dog training while the dogs were engaged in a program of maintenance training for detector dog work. We hypothesized that the correlation between resting-state FC in the dog brain and behavior measures would significantly change during their detection training process (from TP1 to TP2) and would maintain for the subsequent several months of detection work (from TP2 to TP3). This was based on the premise that detection training would lead to strengthening of certain functional connectivities from TP1 to TP2, primarily in brain regions/networks involved in processing of reward cues, reinforcement learning and olfactory processing. Behaviorally, we observe that these functions are engaged during detection training and improve from TP1 to TP2. In addition, whatever gains are made during detection training are often maintained behaviorally from TP2 to TP3 and this is referred to as the maintenance period. Maintenance of learned detection-related behaviors is critical for the operational effectiveness of these dogs. Consequently, we hypothesize that strengthened functional connectivity in the above networks would then be maintained from TP2 to TP3. Further, the strengthening of functional connectivity in identified paths would also mirror corresponding behavioral improvements from TP1 to TP2 and subsequent maintenance till TP3. The underlying assumption is that improvements in behavior are supported by strengthening of connectivity in brain regions/networks involved in essential aspects of detection training, i.e., the processing of reward cues, the reinforcement learning, and olfactory processing. This assumption is supported by the human neuroimaging literature showing a significant correlation between gains in task performance with increased functional connectivity in regions subserving the task [36]. The study aims to explore whether brain connectivity patterns measured using fMRI can predict a dog’s success in a training program. By examining brain activity before training, we hope to identify biomarkers that could be used to select dogs more likely to become effective working dogs.

## 2. Materials and Methods

We recruited forty Labrador retriever dogs (24 males/16 females) with ages in the range of 12 to 36 months from the Auburn University Canine Performance Sciences Program and iK9 LLC (www.ik9.com accessed on 29 February 2024).

### 2.1. Dog Training and Preparation

The dogs for this study came from a working dog acquisition process intended to select dogs that have the potential to be trained successfully for working tasks. A standardized assessment test was used for judging the workability of candidate dogs, and that assessment was also used as a behavioral measure for comparison with fMRI imaging metrics.

**Figure 1:**
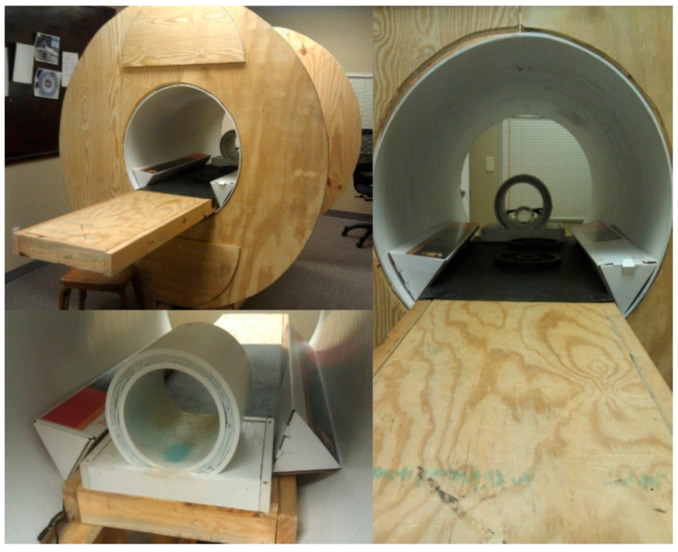
Mock MRI scanner and mock head coil for training dogs.

Once acquired, the dogs began training for being scanned in the MRI while fully awake and unrestrained. For this purpose, a full-scale MRI simulator was fabricated. Additionally, a couple of simulated human knee coils, into which the dog must learn to place and hold the head, were fabricated for use in training the MRI routine (Figure 1). To prevent any possible hearing damage, the dogs wore suitable ear protection during the procedure.

**Figure 2:**
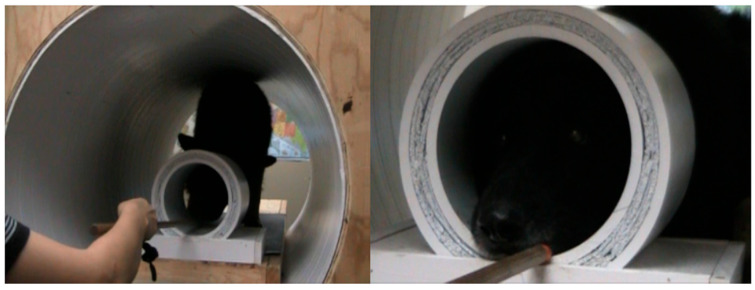
A dog in the MRI simulator being prompted to place his head in the mock coil.

The training process was based on progressive positive principles reinforcement learning and was separated into two stages. Throughout the first stage of the training process, a recording of the MRI operation sound was played, and the volume of the sound gradually increased until it was similar to an actual scan. Once a dog put its head within the knee coil (Figure 2) and remained relatively motionless for approximately 5 min, it was treated with food rewards. When the dog repeated this performance several times across the course of an approximately 30 min training session, they were ready for the next stage.

The second stage of training was performed inside the real MRI scanner with the running of a functional sequence. Transitioning to the actual MRI went smoothly for some dogs but was more difficult for others. The final target performance for the training was for a dog to voluntarily enter the MRI scanner, position its head into the knee coil and remain relatively motionless for an approximately 5 min run and repeat the behavior across multiple runs within an hour-long session of scanning. The time to train the dogs from initial training to successful scan in the actual MRI ranged from 12 h to 30 h (on average, about 18 h), which was divided into several one-hour sessions across days. More details about the training procedure can be obtained from our previous publications [8,21,22,23,37].

### 2.2. Longitudinal Experimental Design

**Figure 3:**
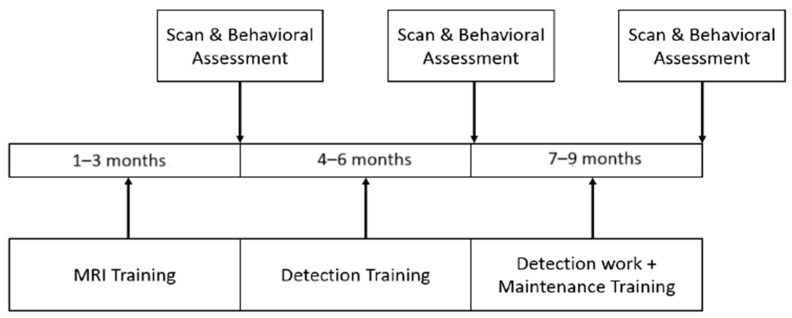
A schematic of the longitudinal experimental design.

To track the changes in functional imaging metrics with time, all of the fMRI scans and behavioral measures were acquired at multiple time points (TPs) across the time of participation of the dogs in this study (Figure 3). The first time point (TP1) was prior to the dogs entering formal working detector dog training, but after 1–3 months of MRI training to keep their head still inside the scanner. The second time point (TP2) was soon after formal detector dog training which lasted about 3 months. The third time point (TP3) was three months post detector dog training while the dogs were engaged in a program of maintenance training for detector dog work.

### 2.3. Working Dog Assessments

All of the dogs in this project were assessed for their potential to be successfully trained and employed for working detector dog tasks. The assessment we employed is a variant of those widely used across many operational agencies (e.g., U.S. Military, Homeland Security agencies, law enforcement agencies) for assessing candidate working dogs. The assessment has two components, performance, and environmental soundness [38,39]. The performance element assessed the propensity of the dog to retrieve a thrown object; interest, focus, and desire to possess a toy reward; the propensity of the dog to use its nose in hunting for a desired object; the amount of effort and degree of distractibility during retrieving and hunting games. Utilizing thrown objects in training exercises enables dogs to hone their olfactory abilities by searching and recognizing a specific scent. Throwing objects can also serve as a reward-based training method since after successfully retrieving the target objects, a dog could be rewarded with a treat or praise. This type of positive reinforcement can help to motivate the dog to continue to work hard and improve its detection skills. Similarly, engaging in hunting activities allows dogs to exercise their cognitive abilities by using a range of problem-solving, critical thinking and decision-making skills. Additionally, hunting also helps dogs to enhance their physical abilities such as agility and endurance, which also can be useful for detection work. The environmental soundness element assessed the extent of startle and ability to recover from sudden loud noises; comfortableness with and ability to overcome initial difficulty with novel surfaces (such as slick floors), obstacles (such as open stairs), and surroundings; and reaction to strange/new persons, places, and busy urban settings. All these activities can help to sharpen dogs’ detection skills, including their sense of smell, decision-making, focus, and commitment. A composite score from 1 (low proficiency) to 5 (high proficiency) was assigned for each of the measures: retrieve, hunt, and environmental soundness. The scores for all three measures were summed to provide one integrated working dog assessment score named “Integrated Behavioral Score”. This measure was then used for correlation with imaging metrics.

### 2.4. Data Acquisition

Functional MRI data were acquired using a T2*-weighted single-shot echo planar imaging (EPI) sequence on a Siemens 3 Tesla Verio scanner (Erlangen, Germany) with 16 axial slices, slice thickness = 3 mm, repetition time (TR) = 1000 ms, echo time (TE) = 29 ms, field of view (FOV) = 150 × 150 mm^2^, flip angle (FA) = 90 degree, in-plane resolution 2.3 × 2.3 mm, in-plane matrix = 64 × 64, and 200 temporal volumes in each run. Two resting-state runs were acquired for each dog at each time point. A 15-channel human knee coil was used as a head coil for the dog brain, and all dogs were trained to keep their heads in the coil as still as possible (with eyes open) during the scanning. Anatomical images were acquired using T1-weighted, 3-dimension magnetization-prepared rapid-gradient echo (3D-MPRAGE) sequence for overlay and localization (TR = 1990 ms, TE = 2.85 ms, FA = 9 deg, FOV = 152 × 152 × 104 mm^3^, in-plane matrix = 192 × 192, number of partitions = 104, for voxel size = 0.8 × 0.8 × 1.0 mm^3^). All MRI scans included generalized autocalibrating partially parallel acquisitions (GRAPPA) with an acceleration factor = 2.

As described in our previous publication [22], we were able to acquire good-quality data from the frontal regions of the brain (which are susceptible to distortions) as well. As described in our previous publications, we employed a single-camera-based external optical head motion tracker [8,21,23] during data acquisition. This device provides motion estimates with a high spatiotemporal resolution and was used in conjunction with realignment parameters to assess the quality of the data. When we detected motion amounting to more than one voxel from the external camera, the data were completely discarded with the assumption that even motion censoring will not be able to retrieve usable data. When we detected motion amounting to less than one voxel from the external camera, framewise displacement was estimated using the acquired data post hoc and motion censoring and interpolation of discarded volumes was employed when framewise displacement exceeded 0.2 mm. As part of preprocessing, the realignment of scans was used to estimate 6 motion parameters for each subject (3 translation parameters and 3 rotation parameters). Afterwards, the framewise displacement (FD), an overall measure of motion, was computed by using these parameters [40]. The threshold was selected based on several literatures [15,21,22].

The longitudinal training and assessment process (Figure 3) was performed in 40 dogs. However, due to the relatively long period of time that the dogs were expected to be in the longitudinal training and assessment process, some dogs had to be released from imaging at different time points. Taken together with data discarded due to motion, we had to reject data from 10 dogs in TP1, 14 dogs in TP2, and 16 dogs in TP3. Thus, usable data from all the time points included a total of 154 scans/runs from 30 dogs, with 60 scans from 30 dogs (17 males/13 females) at TP1, 49 scans from 26 dogs (14 males/12 females) at TP2, and 45 scans from 24 dogs (14 males/10 females) at TP3. Only dogs which had usable data at all three time points (i.e., 24 dogs) were analyzed in the current study. The data were incorporated into the “Connectivity–Behavior Correlations across Timepoints” section in the study. For the predictivity analysis, 30 dogs from TP1 were selected with 13 assessed as suitable for detection work (7–9 months assessment).

### 2.5. Image Preprocessing

The preprocessing of raw RS-fMRI data was performed using SPM12 and the DPARSF toolbox [41,42]. The preprocessing steps included slice-timing correction, realignment to the first functional image (i.e., image-based correction for head motion by aligning 3D volumes acquired at different time-points), spatial normalization, spatial smoothing with a Gaussian kernel of 4 × 4 × 4 mm^3^ full width at half maximum (FWHM), detrending and temporal band-pass filtering (in the range 0.01–0.1 Hz) for removing low- and high-frequency sources of noise. Further, variance due to nuisance factors such as the six head motion parameters (3 translations and 3 rotations), motion parameters obtained from the external camera, as well as white matter and cerebrospinal fluid signals were regressed out from each voxel time series inside the dog brain. Unlike human experiments, spatial normalization in dogs is not straightforward due to the lack of a general template such as the MNI template in humans. Existing templates for dogs are derived from less than ten dogs and thus may not capture the head size variability across different breeds. Therefore, we used a relatively more accurate two-step spatial normalization method, which was employed in our previous dog fMRI studies [8,21,22,23,37]. In short, firstly, a good-quality template was chosen among a pool of dogs (dogs in an anesthetized state are ideal to minimize moments) in our previous studies [21,22]. Then, anatomical images of all the subjects were co-registered in that template and the functional images of all subjects were co-registered to their respective anatomical images. We could then transition from the functional images to the selected ones using these steps.

### 2.6. Characterization of Resting-State Brain Networks

Resting-state networks are defined as the collection of brain regions that are temporally correlated with each other. In the literature, two predominant approaches have been employed for characterizing resting-state networks: seed-based connectivity and connectomic approaches. In the seed-based method, networks are defined based on the strength of correlation between a seed region and every other region of the brain. Consequently, many networks can be defined based on different seed regions and these networks have shown to be correlated with human personality and behavior [43,44,45]. For example, the default mode network (DMN) has been shown to correlate with traits and capabilities [46,47], and the reward network (RN) has been shown to be correlated with reward and reinforcement learning in humans [43]. However, seed-based connectivity obtained from predefined ROIs is limited by the fact that they do not capture interactions between all brain regions. In order to alleviate this limitation, connectomic approaches have been proposed as a more holistic measure which captures functional associations between all possible pairs of brain regions simultaneously [48]. Therefore, region-wise resting-state FC was obtained using Pearson’s correlation between regions taken pair-wise across the entire brain. The regions themselves were identified by those authors with expertise in canine brain anatomy using a previously published dog atlas as a guide (http://vanat.cvm.umn.edu/mriBrainAtlas/ accessed on 29 February 2024). For example, if there were N regions inside the brain of a given dog, then this would result in an *N* × *N* region-wise connectivity matrix for that dog. These connectivity matrices were further used in analyses described below.

### 2.7. Connectivity–Behavior Correlations across Timepoints

**Figure 4:**
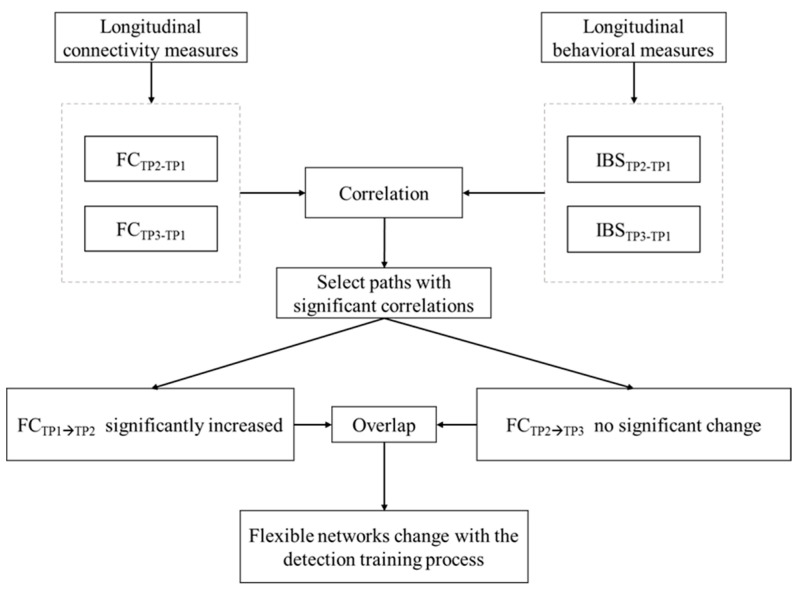
A schematic of the two steps connectivity–behavior correlation analysis.

We hypothesized that the correlation between resting-state FC in the dog brain and behavior measures would significantly change during their detection training process (from TP1 to TP2), and would maintain for the subsequent several months of detection work (from TP2 to TP3). This was achieved by two steps (Figure 4): (1) the difference in the FC for each subject and the path between TP1 and TP2 were correlated with the difference in the corresponding behavioral measures between TP1 and TP2, respectively. This yielded paths whose resting-state connectivity differences FC_TP2-TP1_ significantly correlated (*p* < 0.05, uncorrected) with corresponding differences in the integrated behavioral score IBS_TP2-TP1_. (2) Among paths satisfying this condition, we retained those paths whose FC significantly increased (*p* < 0.05, uncorrected) from TP1 to TP2, but did not change significantly from TP2 to TP3. This was based on the premise that detection training would lead to the strengthening of certain functional connectivities from TP1 to TP2, which would then maintain those FC levels from TP2 to TP3 during maintenance training. Further, the identified paths would also mirror corresponding behavioral improvements from TP1 to TP2 and subsequent maintenance to TP3. Given our expectation that the significant paths identified between TP1 and TP2 will remain consistent between TP2 and TP3, it is worth examining if any differences in FC paths between TP1 and TP3 (FC_TP3-TP1_) are also significantly correlated with behavioral changes between TP1 and TP3 (IBS_TP3-TP1_), hence supporting our hypothesis. The resting-state brain network resulting from this analysis represents the flexible networks which change with the detection training process.

### 2.8. Brain Networks Predictive of Dogs’ Suitability for Detection Work

Networks that statistically correlate with behavioral changes across time do not necessarily guarantee their ability to predict which dogs are suitable for detection work using pre-training data at TP1. To further study the resting-state FC features that can predict the success of training, dogs at TP1 were divided it two groups: the successful group consisting of 13 dogs which were eventually deemed suitable for detection work at the end of the longitudinal training and assessment process (7–9 months post-recruitment, Figure 3) and a non-successful group consisting of 17 dogs which were eventually deemed unsuitable for detection work. FC paths that were significantly stronger (*p* < 0.01) in the successful group compared to the non-successful group at TP1 were determined and used as input features to the classifier. This could enhance the quality of classification and ensure that non-discriminatory features are not fed into the classifier.

In order to determine classification accuracy, i.e., the ability of FC features from TP1 identified above to predict whether a given dog would eventually fail or succeed, logistic regression was used as the training kernel since it performed consistently well. Since this is a popularly used classifier, we have skipped including a description of it and we refer to previous work for a detailed description of its underlying principles [49]. Also, using this classifier allowed us to compare our results with a previous study which is similar to ours [26]. The receiver operating characteristic (ROC), which plots the true-positive rate (TPR) against false-positive rate (FPR) and thus does not depend on a specific threshold, was generated and the area under the curve (AUC) for the ROC was used as a metric for performance evaluation. Four-fold cross-validation was employed so that the model could be built using training data and then be tested using validation data. This minimizes the chances of model overfitting.

In order to better understand the functional roles of paths (and corresponding regions) that correlated with behavioral changes due to detection training, we identified the homologous regions (strictly functionally analogous regions) between dogs and humans by comparing the similarity of connectivity fingerprints of these regions. We manually selected 154 human subjects to match the number of scans, gender, and age in dog year equivalents based on Lebeau’s model [50] with our dog group. For more details about data acquisition and preprocessing of the human data, please refer to previous publications [51]. To establish connectivity fingerprints for each subject, we predefined 19 “targets” which were ROIs covering most of the cortical regions as well as several subcortical regions (Figure S1, Table S2) that are known to play a crucial role in guiding canine behavior as borne out by previous literature [52]. Detailed analysis is available in the Supplementary Materials.

All identified connectivity paths were mapped onto the brain surface using BrainNet Viewer software (v.1.41) [53].

## 3. Results

**Figure 5:**
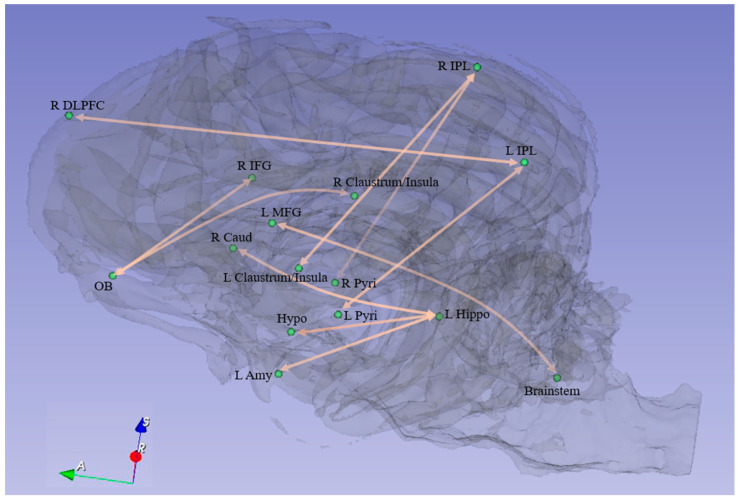
Functional connectivity paths in the dog brain whose FC values satisfied our hypotheses. Pyri = pyriform, IPL = inferior parietal region, Hippo = hippocampus, Amy = amygdala, Hypo = hypothalamus, MFG = middle frontal region, Caud = caudate, OB = olfactory bulb, DLPFC = dorsolateral prefrontal cortex, IFG = inferior frontal region. R and L correspond to right and left brain hemispheres, respectively.

**Figure 6:**
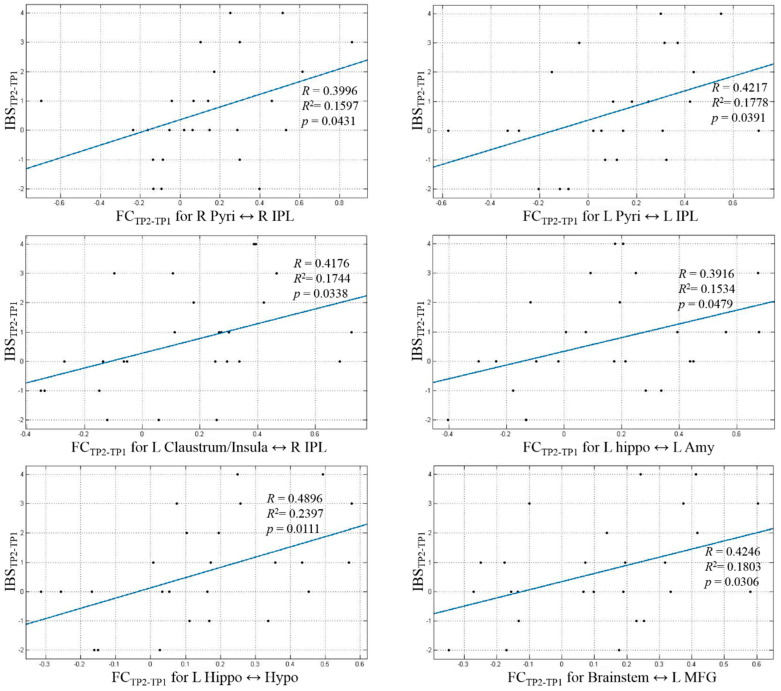

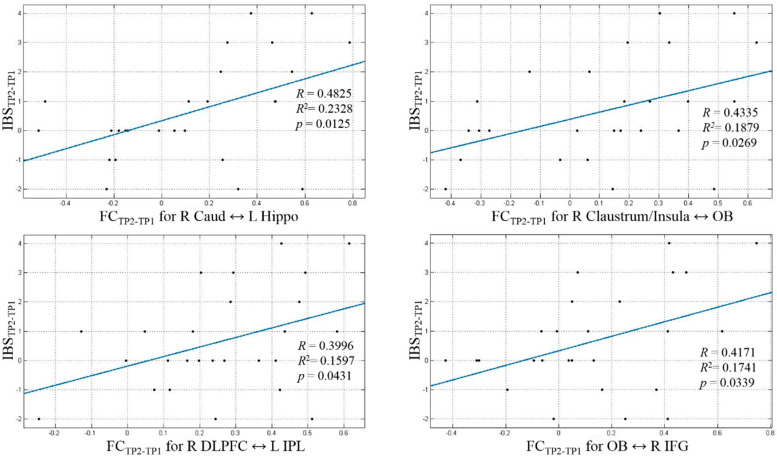
Resting-state connectivity differences between TP2 and TP1 (FCTP2-TP1) in the dog brain significantly correlated (*p* < 0.05, uncorrected) with corresponding differences in the integrated behavioral score IBSTP2-TP1.

**Figure 7:**
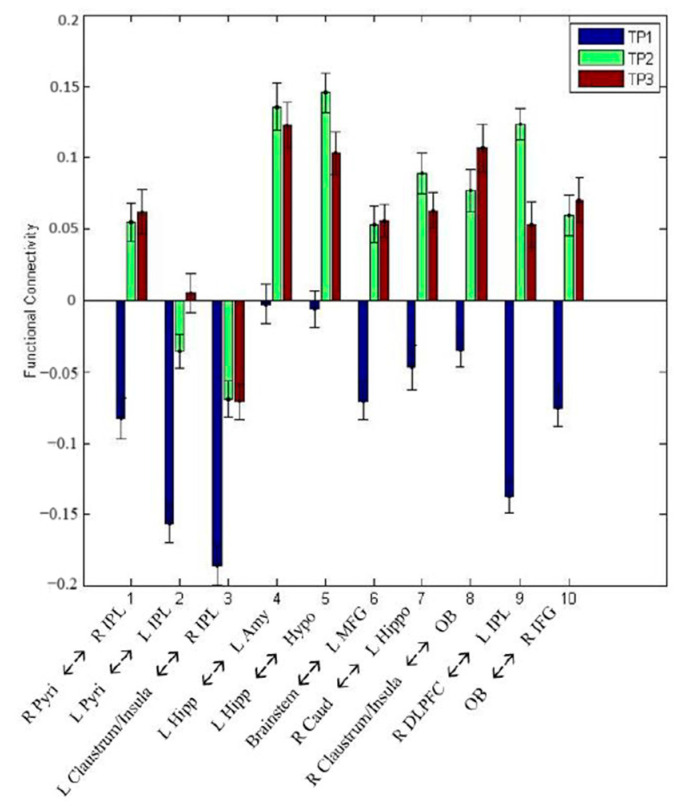
FC paths in the dog brain at each time point whose strength significantly increased (*p* < 0.05, uncorrected) due to detection training (from TP1 to TP2) and then maintained from TP2 to TP3 (*p* > 0.05).

The IBS behavioral score increased significantly from TP1 to TP2 (*p* < 0.05) and did not change significantly from TP2 to TP3 (*p* > 0.05). Also, the difference in scores between TP1 and TP2 (i.e., TP2-TP1) was significantly (*p* < 0.05) larger than the corresponding difference in scores between TP2 and TP3 (i.e., TP2-TP3). We identified ten paths in accordance with our hypotheses (Figure 5 and Table 1). These paths satisfied two different criteria. First, resting-state connectivity difference FC_TP2-TP1_ significantly correlated (*p* < 0.05, uncorrected) with corresponding differences in the integrated behavioral score IBS_TP2-TP1_ (Figure 6). Second, the strength of these paths significantly increased (*p* < 0.05, uncorrected) from TP1 to TP2 and then maintained from TP2 to TP3 (*p* > 0.05) (Figure 7 and Table 2). We did not find any paths that weakened their connectivity strength from TP1 to TP2 and TP3. Further, the difference in connectivity between TP1 and TP2 (i.e., TP2-TP1) was significantly (*p* < 0.05) larger than the corresponding difference in connectivity between TP2 and TP3 (i.e., TP2-TP3). This demonstrates that detection training would lead to the strengthening of certain functional connectivities from TP1 to TP2, which would then maintain those FC levels from TP2 to TP3 during maintenance training. The “strengthening” of these paths involved activity in corresponding ROIs coming in phase (as seen from the positive correlations) in TP2 and TP3 as compared to being out of phase in TP1 (as evidenced by negative correlations in TP1). Further, the identified paths mirrored corresponding behavioral improvements from TP1 to TP2 and subsequent maintenance at TP3.

### 3.1. Successful vs. Non-Successful Working Dogs

**Figure 8:**
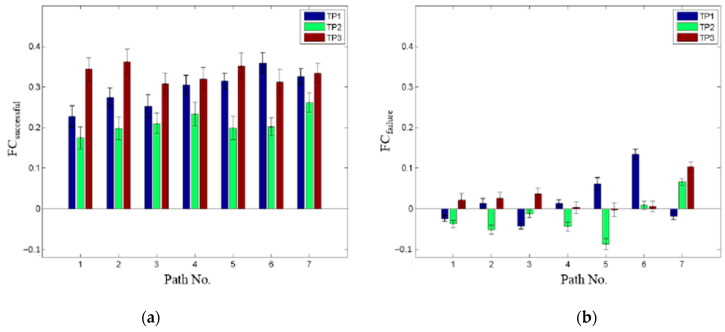
FC paths in the dog brain whose strength was significantly stronger in the successful group (**a**) as compared to the non-successful group (**b**) specifically at TP1, TP2, and TP3.

**Figure 9:**
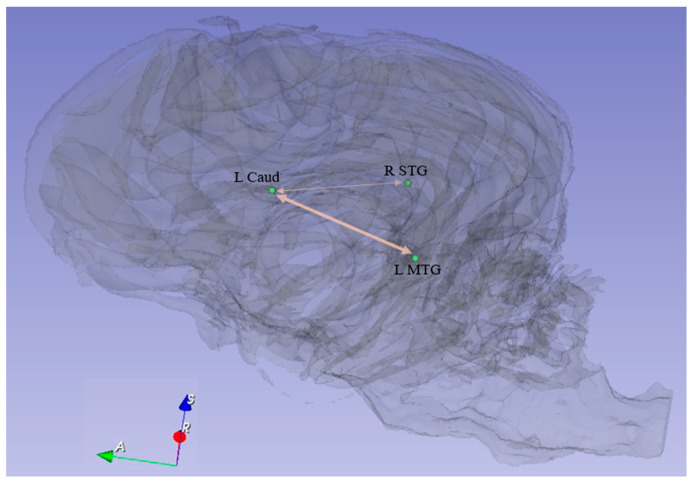
A pictorial spatial representation of the paths in the dog brain shown in Table 3. The thick line corresponds to multiple paths between L Caud and L MTG while the thin line corresponds to the single connection between L Caud and R STG. Caud = Caudate, MTG = middle temporal region, STG = superior temporal region. R and L correspond to right and left brain hemispheres, respectively.

We identified seven paths in the dog brain (Table 3, Figure 8) whose FCs were significantly stronger in the successful group (n = 13) as compared to the non-successful group (n = 17) at TP1, TP2, and TP3 (Figure 9), but did not change with training (Table 4). Among them, six paths were located between Caudate and L MTG.

### 3.2. Classification Analyses

**Figure 10:**
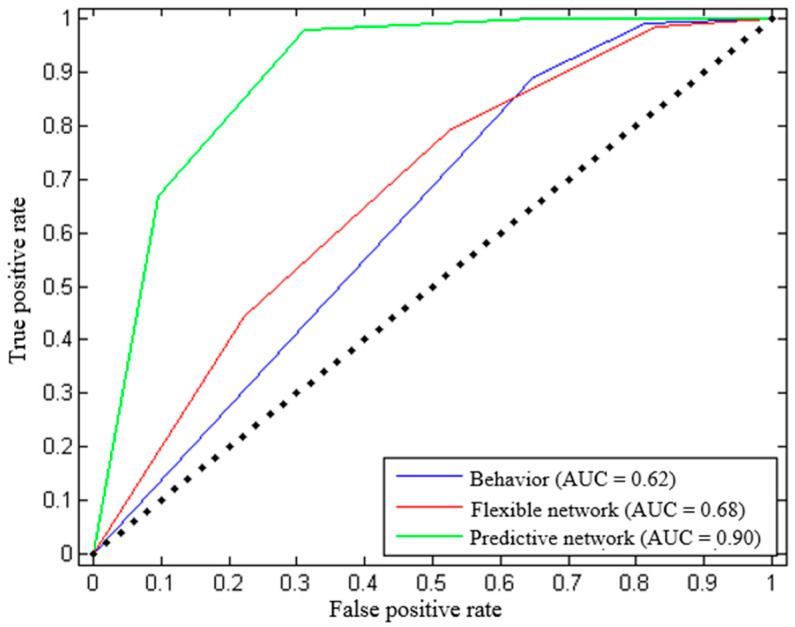
ROC plot for the three classifier models used to predict, using data at TP1, whether a dog would eventually be suitable for detection work. The straight line in the AUC curve is a reference line indicating random performance. A classifier’s ROC curve should lie above this line to be considered useful for classification tasks.

Considering that the sample size was relatively small, we performed classification analyses using 1000 iterations of stratified random shuffling with a test size of 25% of the data (four-fold cross-validation). Classifiers with behavior (integrated behavioral score) performed above chance with AUC equal to 0.62 (Figure 10, blue). Classifiers using functional connections in a flexible neural network in the dog brain which changed with detection training and correlated with corresponding behavioral changes (Table 1) also performed above chance with AUC = 0.68 (Figure 10, red). However, the best classification performance was achieved using functional connections within a network of regions in the dog brain which was significantly stronger in the successful group as compared to the non-successful group (Table 3) but did not change with training (Table 4). This network gave an AUC equal to 0.90 (Figure 10, green).

## 4. Discussion

Dogs have a unique ability to interact with humans and this ability has led to dogs working with and assisting humans in various tasks. Therefore, investigations on their general behavioral capabilities and their related neural bases can inform us about critical parameters for selecting dogs for training. However, research into the neural basis of the behavior of dogs, specifically longitudinal investigations, is sparse. This study is the first to our knowledge to explore the neural processes across different training time points at in vivo level using resting-state fMRI.

Previous studies have shown the reconfiguration of brain networks during task learning in humans [54,55,56,57]. Such regional network dynamics are consistent with a core–periphery model wherein certain brain regions show relatively stable (with respect to time) patterns of interaction that are necessary for the task performance, while others display relatively flexible patterns that support learning and changes in task performance [55]. In our work, we extended this concept to the dog training process and hypothesized that such a core–periphery network may not only mirror changes in behavior with training, but also predict the success of dog training and could potentially be used for selecting dogs to be trained.

We identified two systems, one system (the periphery system) consisting of a brain network which strengthened its connectivity with improvements in canine behavior scores after detection training. We believe that this is a flexible system which supports learning related plasticity during the training process. However, the periphery system is not predictive, and its connectivity at the first baseline time point prior to training could not predict whether a given dog could eventually be trained to become a good detector dog. The other system we identified did not show significant changes in its connectivity strength during the training process. However, the strength of connectivity of this core network predicted, with 90% accuracy, whether a given dog would eventually graduate as a detector dog from the training regimen. We speculate that the core stable network may be an endophenotype that is inherited and mainly controlled by genes while the flexible periphery network may be amended by environmental influences [58,59]. Below, we discuss this core–periphery model in greater detail.

### 4.1. Flexible Periphery Network Underlying Detection Training

**Figure 11:**
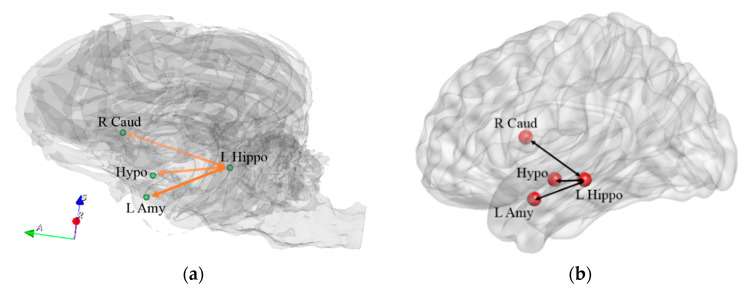
Olfaction-related network in the dog brain (**a**) and homologous regions in the human brain (**b**) which showed significant correlations between behavioral changes and connectivity changes. Hippo = hippocampus, Amy = amygdala, Hypo = hypothalamus, Caud = caudate. R and L correspond to right and left brain hemispheres, respectively.

**Figure 12:**
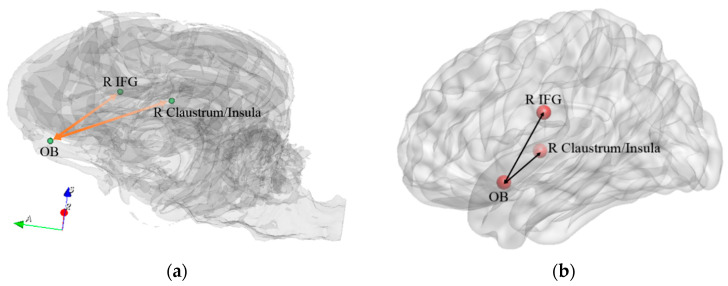
Positive reinforcement network in the dog brain (**a**) and homologous regions in the human brain (**b**) which showed significant correlations between behavioral changes and connectivity changes. OB = olfactory bulb, IFG = inferior frontal region. R and L correspond to right and left brain hemispheres, respectively.

We found significant correlations between behavioral changes and connectivity changes between the following ROIs related to olfactory processing: L Amy and L Hippo, L Hippo and Hypo, and L Hippo and R Caud (Figure 11). Previous human studies [60,61,62] as well as dog studies [21,22] have implicated this set of regions during olfactory processing. Further, previous studies have shown that functional connectivity of olfaction-related networks may be reinforced by training, and training-induced behavioral improvement in olfactory performance has been observed in healthy humans [63]. Therefore, this sub-network may be related to an improvement in the olfactory processing capabilities of dogs during detection training. On the other hand, the insula and inferior frontal gyrus were commonly activated by visual and odor food cue stimulation in humans in a meta-analysis study on food cue neuroimaging [64]. Considering that during the training process, dogs were reinforced for successful performance with treats, the increase in FC between the OB and R insula, as well as between the OB and IFG (Figure 12) might reflect positive reinforcement and the neural plasticity of conditioning for food-related stimuli.

**Figure 13:**
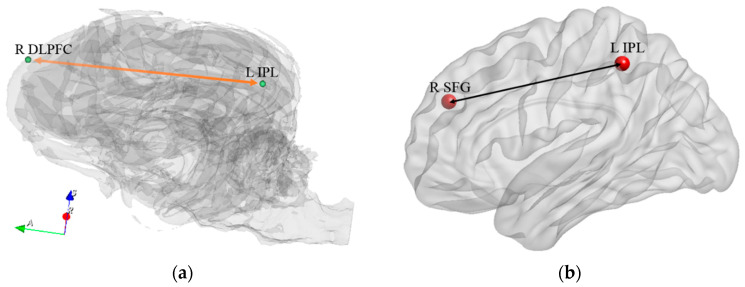
The fronto-parietal network of the dog brain (**a**) and homologous regions in the human brain (**b**) which showed significant correlations between behavioral changes and connectivity changes. DLPFC = dorsolateral prefrontal cortex, IPL = inferior parietalregion, SFG = superior frontal region. R and L correspond to right and left brain hemispheres, respectively.

Paths from the R SFG to L IPL in the periphery network may be part of the fronto-parietal network (Figure 13). This is consistent with human studies that have shown that behavioral variables co-vary with connectivity in frontal-parietal networks (FPN) [12,14]. Previous human studies have suggested that brain regions in the frontal and parietal cortices play an important role in cognitive control processes and connectivity within the FPN directly relates to attention [65,66,67]. An ICA study has shown a greatly overlapped frontal-parietal network in macaque and human brains, suggesting an evolutionary preserved frontal-parietal system [68]. Moreover, studies related to individual human intelligence found that greater connectivity, especially during task performance, within the frontal-parietal network was associated with higher intelligence scores [69,70].

**Figure 14:**
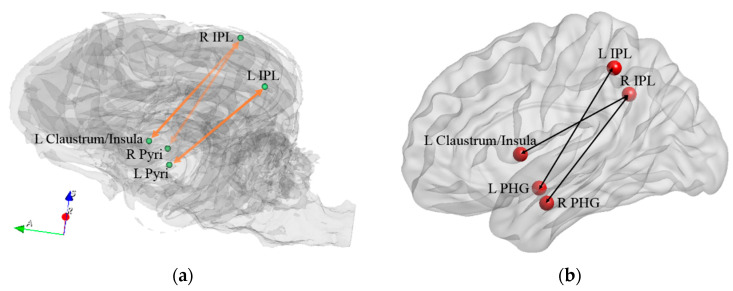
The network related to familiarity and recollection in the dog brain (**a**) and homologous regions in the human brain (**b**) which showed significant correlations between behavioral changes and connectivity changes. PHG = parahippocampal gyrus, IPL = inferior parietal region, Pyri = pyriform. R and L correspond to right and left brain hemispheres, respectively.

PHG and insula are also known to be involved in familiarity-related judgments [71,72,73]. Also, the IPL is known to be associated with familiarity and recollection-related judgments [74]. The increase in the FC within the FPN, between the L insula and IPL, as well as between the PHG and IPL (Figure 14) with corresponding improvements in behavior might suggest improved understanding and reaction towards the trainer’s gestures and commands (via both familiarity and recollection) through learning.

**Figure 15:**
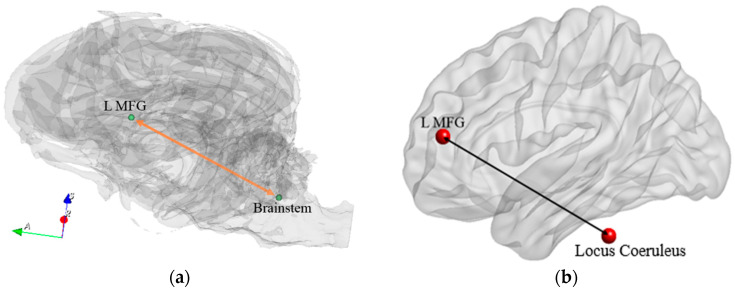
Learning-related network in the dog brain (**a**) and homologous regions in the human brain (**b**) which showed significant correlations between behavioral changes and connectivity changes. MFG = middle frontal region. R and L correspond to right and left brain hemispheres, respectively.

The Locus coeruleus (LC) in the brain stem is the largest repository of Norepinephrine (NE) in the human brain [75]. Noradrenergic neurons within LC are widely distributed and are one of the main ascending pathways from the LC projects to the prefrontal cortex [76,77]. It has been shown that NE projections from the LC to the cortex support learning and memory retrieval [78,79]. An animal study has further found that boosting NE transmission can lead to increased functional connectivity [80]. Thus, the significantly increased FC (from baseline time point to other TPs post detection training) between the LC in the brainstem and L MFG (Figure 15) might correspond to the mechanisms of learning of odors and the retrieval of such memory.

### 4.2. Stable Core Network for Predicting Training Outcomes

It can be noted that a majority of the paths in the stable core network involve the caudate. Previous studies have considered the caudate as a part of the reward system and may reinforce learning in humans as well as dogs [22,30,43]. Studies in humans and monkeys have indicated that regions in the temporal cortex respond preferentially to face recognition [81]. Further, a recent canine study has shown that using fMRI activations from the caudate, amygdala, and a specialized region in the temporal cortex for face processing (known as the dog face area or DFA), the authors were able to predict (through cross-validation) a given dog’s suitability for assistance work with an accuracy of 80% [28]. Thus, it is not surprising that we found multiple paths between the L Caud and L MTG in the stable core network that were stronger in the successful group at TP1. Further, the strength of connectivity of these paths at TP1 was able to predict the success of training with an accuracy of 90% using a classifier with cross-validation comparable to the one used by Berns et al., 2016 [28].

The main role of the STG is to process sound stimuli [82]. During the training process, dogs were reinforced for successful performance with treats and verbal rewards (the trainer praised the dog—“Good dog” or “Yes”). Stronger connectivity between the L Caud and R STG in the successful group might suggest that the dogs that were able to associate human verbal praises with rewards might have a better chance to be trained as detection dogs. One study [83] shows the significant role of human voices in shaping canine emotional experiences, revealing parallels in the dogs’ reactions to basic emotional expression in human vocalizations like those observed in humans. This indicates a profound interplay between human speech patterns and canine emotional comprehension.

It should be noted that the caudate was found to be a part of both the core and periphery networks (L Caud in the stable core network and R Caud in flexible periphery network). The factor that prohibits dogs from successful training is believed to be their fearfulness/anxiety towards novel/complex environments [84]. Previous studies have suggested that dopamine D4 receptors influence canine fearfulness, anxiety and impulsivity related traits [85,86]. Dopamine D4 receptors have been found to be concentrated in the caudate [87]. Since the expression of D4 receptors in the caudate is controlled by specific genes, they might influence fearfulness-, anxiety-, and impulsivity-related traits in dogs, thereby influencing baseline neural connectivity between the caudate and other brain regions. This might be a potential mechanism by which the caudate may be involved in the stable core network that predicts whether dogs can be trained to become successful working dogs. On the other hand, the role played by the caudate in reinforcement-based learning in humans is known [88,89]. Specifically in dogs, Berns et al. found that the caudate was significantly more active when the dogs were exposed to different types of reward stimuli [26,27,28,30,90]. We found an increase in FC between the R caudate and L hippo in the periphery network with detection training. Therefore, our findings also support the role of the caudate in learning, reward, and training-related neural plasticity.

Taken together, we identified a core-periphery organization in the dog brain and these systems responded differently to the detection training process, which appear to represent ontogeny and phylogeny. The flexible periphery network is ontological, as it changed with corresponding behavioral changes due to training/learning that were mainly located in regions implicated in odor processing. Biologically, network flexibility might be driven by physiological processes that facilitate the participation of corresponding regions in multiple functional communities while learning new tasks. On the other hand, the caudate-based core network is driven more by phylogeny because of its stability across detection training. Such a stable network may contain information about intrinsic learning ability for individuals which can successfully predict the outcome of training. Our result suggests that, upon replication and refinement, fMRI-based resting-state brain connectivity may assist in choosing dogs that are more easily trainable for performing detection tasks before they enter the training regimen. They also suggest that good working dogs learn efficiently, and they can be trained well in what we want them to learn.

### 4.3. Insights from Human Homology

A basic challenge in animal neuroimaging is to compare and explain brain functions across species, especially at the voxel level. Interpretation often relies on the assumption that putative homologous areas are functionally similar [91]. However, this assumption is not always valid [92] since putative homology is generally not established on any rigorous statistical premise. Previous studies have suggested that specific functions in an area of one species may be shifted to other regions in other species [93]. In this study, we sought to identify the homologous areas by comparing the connectivity fingerprints of regions between humans and dogs [94,95]. This was carried out because there is relatively abundant literature about the functional roles of brain regions in the human brain than in the dog brain. The approach of matching connectivity fingerprints is a viable technique and has been used in a number of studies previously [96,97].

We identified several regions that do not functionally correspond to their putative homologous regions. For example, the R pyriform, L pyriform, and R dorsolateral prefrontal cortex in dogs shared similar connectivity profiles as the R parahippocampal gyrus, L parahippocampal gyrus, and R superior frontal gyrus in humans. Moreover, the LC in the human brainstem was identified to be homologous to a specific region of the dog brainstem. With the help of previous human literature and based on the homology established using the connectivity fingerprint matching procedure, we speculated that the connectivity between the brain stem and the frontal cortex in dogs corresponded to that between the LC and L MFG in humans and this might underlie mechanisms of learning of odors and retrieval of such memory. Despite the lack of abundant literature into the neural basis of behaviors of dogs and their trainability, projecting dog brain regions identified here onto the human brain and evaluating similar literature on humans may help us better understand the evolutionary role of brain–behavior relationships. This comparative evaluation is particularly relevant for dogs since they are a rare species which has socially co-evolved with humans for thousands of years.

## 5. Limitations and Future Research

A few limitations of this work are noteworthy. Although the number of dogs is comparatively large, the sample size is still considered small in an absolute sense which did not allow us to use stringent p-value thresholds that would reduce the false-positive rate. Given that the field of canine imaging is in its infancy, we believe that standards applicable to human imaging should not be applied to canine imaging at this point in time. Once procedures used for training dogs to keep their head still inside the scanner are mastered and a workforce with those skills is developed, it will become feasible to increase the sample size in the future and that will allow more conservative statistical thresholds to be used. Until that happens, accepting a higher false-positive rate in canine imaging results will nurture discovery science in this nascent field. For example, the identified dog core-periphery model could provide a window into the evolutionary role of brain–behavior relationships and provides great potential for answering questions about phylogeny and ontogeny in future studies. It should be noted that no significant gender differences were found in the identified two systems in our study; however, future studies should aim to replicate our findings in different breeds of dog. Further, we have compared our classification results with those from a previous study [28]. However, here we studied detection dogs while they focused on service dogs. Therefore, the comparison of our results with those reported by Berns et al. is qualitative at best. As the study is exclusively on labradors, it is important to note that the findings may not be applicable to other breeds of dogs due to distinct genetic and physiological characteristics that vary between different breeds. Therefore, we should consider diversity in the dog population and include a broader range of breeds in the future studies to increase the generalizability of the findings.

This study’s behavioral assessments were conducted over a relatively short period, while prior research has highlighted the importance of longer intervals for observing meaningful variations in the scores [39]. To evaluate the consistency of behavior over an extended period, using a longer interval of assessment may be necessary. In addition, in a study that tracks individuals over an extended period, the lack of control for maturation can impede the accuracy of determining cause-and-effect relationships between study variables [98]. Therefore, to achieve more reliable results, we should include a control group that does not participate in the training and take age into account as a potential confounding variable.

## 6. Conclusions

In this paper, through the analysis of resting-state fMRI data collected from dogs undergoing training for detection tasks in different time points, we have identified core brain regions that may aid in the early prediction of dogs’ success in training. Additionally, our investigation into shared brain regions and functions between humans and canines significantly contributes to our understanding of interspecies cognition and behavior. These findings highlight the promising avenues potential for future research in this area.

## Figures and Tables

**Table 1:** Functional connectivity paths in the dog brain whose FC values satisfied our hypotheses (i.e., increased significantly from TP1 to TP2 and did not change significantly from TP2 to TP3). Pyri = pyriform, IPL = inferior parietal region, Hippo = hippocampus, Amy = amygdala, Hypo = hypothalamus, MFG = middle frontal region, Caud = caudate, OB = olfactory bulb, DLPFC = dorsolateral prefrontal cortex, IFG = inferior frontal region. R and L correspond to right and brain hemispheres, respectively.

Path No.	Path
1	R Pyri ↔ R IPL
2	L Pyri ↔ L IPL
3	L Claustrum/Insula ↔ R IPL
4	L Hippo ↔ L Amy
5	L Hippo ↔ Hypo
6	Brainstem ↔ L MFG
7	R Caud ↔ L Hippo
8	R Claustrum/Insula ↔ OB
9	R DLPFC ↔ L IPL
10	OB ↔ R IFG

**Table 2:** Differences in *p*-values for the strength of FC across time points for paths shown in Figure 7.

	TP2 > TP1	TP3 > TP1	TP2 ≠ TP3
R Pyri ↔ R IPL	1.49 × 10^−2^	1.76 × 10^−2^	9.12 × 10^−1^
L Pyri ↔ L IPL	1.83 × 10^−2^	5.52 × 10^−3^	4.58 × 10^−1^
L Claustrum/Insula ↔ R IPL	2.66 × 10^−2^	3.06 × 10^−2^	9.75 × 10^−1^
L Hippo ↔ L Amy	2.23 × 10^−2^	3.53 × 10^−2^	8.54 × 10^−1^
L Hippo ↔ Hypo	7.71 × 10^−3^	4.39 × 10^−2^	5.18 × 10^−1^
Brainstem ↔ L MFG	1.69 × 10^−2^	1.42 × 10^−2^	9.65 × 10^−1^
R Caud ↔ L Hippo	2.29 × 10^−2^	4.61 × 10^−2^	6.69 × 10^−1^
R Claustrum/Insula ↔ OB	3.73 × 10^−2^	1.92 × 10^−2^	6.79 × 10^−1^
R DLPFC ↔ L IPL	2.75 × 10^−6^	2.17 × 10^−3^	2.62 × 10^−1^
OB ↔ R IFG	1.57 × 10^−2^	1.51 × 10^−2^	8.72 × 10^−1^

**Table 3:** Paths in the dog brain whose FC values were significantly stronger (*p* < 0.01) in the successful group as compared to the non-successful group at each time point. Caud = Caudate, MTG = middle temporal region, STG = superior temporal region. R and L correspond to right and left brain hemispheres, respectively.

	*p*-Value of FC_successful_ > FC_non-successful_		
Path	TP1	TP2	TP3
1–6. Caudate ↔ L MTG	3.38 × 10^−4^	2.9 × 10^−3^	1.9 × 10^−3^
	7.97 × 10^−4^	2.1 × 10^−3^	1.6 × 10^−3^
	8.55 × 10^−5^	1.6 × 10^−3^	4.1 × 10^−3^
	9.91 × 10^−5^	9.4 × 10^−4^	1.7 × 10^−3^
	2.8 × 10^−3^	1.6 × 10^−3^	1.9 × 10^−3^
	3.7 × 10^−3^	4.2 × 10^−3^	1.8 × 10^−3^
7. L Caud ↔ R STG	3.8 × 10^−6^	1.9 × 10^−3^	4.8 × 10^−3^

**Table 4:** Differences in *p*-values for the strength of FC across time points for paths shown in Figure 8. None of them were significant.

	*p*-Value of Successful Group			*p*-Value of Non-Successful Group		
Path No.	TP1 ≠ TP2	TP1 ≠ TP3	TP2 ≠ TP3	TP1 ≠ TP2	TP1 ≠ TP3	TP2 ≠ TP3
1	5.6 × 10^−1^	2.3 × 10^−1^	9.5 × 10^−2^	7.8 × 10^−1^	5.2 × 10^−1^	4.4 × 10^−1^
2	3.8 × 10^−1^	3.5 × 10^−1^	1.2 × 10^−1^	3.4 × 10^−1^	8.7 × 10^−1^	3.3 × 10^−1^
3	6.4 × 10^−1^	5.7 × 10^−1^	2.9 × 10^−1^	5.1 × 10^−1^	2.4 × 10^−1^	5.1 × 10^−1^
4	4.3 × 10^−1^	8.7 × 10^−1^	4.1 × 10^−1^	3.5 × 10^−1^	8.9 × 10^−1^	5.4 × 10^−1^
5	1.7 × 10^−1^	6.9 × 10^−1^	1.7 × 10^−1^	8.8 × 10^−2^	5.1 × 10^−1^	3.5 × 10^−1^
6	6.2 × 10^−2^	6.2 × 10^−1^	2.6 × 10^−1^	6.3 × 10^−2^	9.3 × 10^−2^	9.6 × 10^−1^
7	3.9 × 10^−1^	9.1 × 10^−1^	4.1 × 10^−1^	1.1 × 10^−1^	6.6 × 10^−2^	5.4 × 10^−1^

## Data Availability

Since this project was funded by the US Department of Defense, we cannot deposit the data in a public repository due to contractual obligations. However, individuals interested in accessing the data can request them from the authors and we can approach DARPA (Defense Advanced Research Projects Agency) to provide permission to share the data on an individual basis.

## Supplementary Materials

The following supporting information can be downloaded at: https://www.mdpi.com/article/10.3390/ani14071082/s1, Figure S1: A pictorial spatial representation of “target” regions in human brain and dog brain; Figure S2: A schematic illustration of the connectivity fingerprint matching approach in our study; Figure S3: A pictorial spatial representation of homologous regions in the human brain identified in Table. S3; Figure S4: A pictorial spatial representation of homologous regions in the human brain identified in Table. S4; Table S1: Matched age for dogs and human subjects; Table S2: Montreal Neurological Institute (MNI) coordinates of “target” regions in humans; Table S3: Regions of the dog brain connected by paths identified above (in Table 1) and corresponding homologous regions in the human brain with their MNI coordinates; Table S4: Regions of the dog brain connected by paths identified above (in Table 3) and corresponding homologous regions in the human brain with their MNI coordinates.
