# Supplementary material for: Two Separate Brain Networks for Predicting Trainability and Tracking Training-Related Plasticity in Working Dogs
Source: Animals (Basel). 2024 Apr 2;14(7):1082. doi: 10.3390/ani14071082 (PMC11010877; doi:10.3390/ani14071082)
Supplement: Supplementary file 1 [file animals-14-01082-s001.zip › animals-2918878-supplementary.pdf]

Identification of Homologous Brain Networks in Humans

In order to better understand the functional roles of paths (and corresponding regions) that correlated with behavioral changes due to detection training, we identified the homologous regions (strictly, functionally analogous regions) between dogs and humans by comparing the similarity of connectivity fingerprints of these regions using a permutation testing framework (readers are referred to the following papers for details regarding this method; Mars et al., 2016; Passingham et al., 2002). Connectivity studies often yield high-dimensional profiles that can be hard to summarize. For example, a connectivity fingerprint obtained from a whole brain voxel wise seed based map can have dimensionality equal to the number of voxels in the brain. On the contrary, the connectivity fingerprints proposed by (Passingham et al., 2002) characterize the connections between the 'seed' region with a selected set of other 'target' regions which are important. In this way, connectivity profiles of a seed region can be characterized in a lower dimension which is also useful in avoiding overfitting. We have utilized this concept in our work. Comparing connectivity profiles obtained from data acquired with different scanning parameters for humans and dogs was achieved by normalizing the data to the maximum connection strength in the brain, such that functional connectivity becomes a metric of relative connection strength. Then, a single mean connectivity profile for both the dog and human samples were obtained separately. Using the Manhattan distance measure (described later), we determined the similarity of different fingerprints. Subsequently, the homology between dog and human "seed" region was examined as described below.

Resting state functional connectivity in humans was determined using preprocessed RS-fMRI data from the Human Connectome Projection (HCP) (HCP 500 Subjects + MEG2 Data Release). This dataset included 3T MRI scans from young healthy adults (age range: 22~35 years). We manually selected 154 human subjects to match the number of scans, gender, and age in dog year equivalents (Table 1) based on Lebeau's model (Patronek, Waters, & Glickman, 1997) with our dog group. The raw functional data obtained from HCP were collected on a Siemens Skyra 3T scanner using multiplexed gradient-echo EPI sequence with slice thickness = 3mm, TR = 720ms, TE = 33.1ms, FA = 52°, FOV = 208×180 mm^2, in-plane

matrix = 104 × 90 and 1200 temporal volumes in each run. For more details about data acquisition and preprocessing of the data, please refer to previous publications (Smith et al, 2013) as well as the "HCP 500 Subjects + MEG2 Data Release" reference manual (https://www.humanconnectome.org/storage/app/media/documentation/s500/hcps500meg2releasereferencemanual.pdf).

Table S1. Matched age for dogs and human subjects

Dogs (months)	Human subjects (years)
12-20	22-25
20-28	25-30
28-36	30-35

To establish connectivity fingerprints for each subject, we predefined 19 "targets" which were ROIs covering most of the cortical regions as well as several subcortical regions (Fig. S1, Table S2) that are known to play a crucial role in guiding canine behavior as borne out by previous literature (Andics et al, 2014, 2016; Berns et al, 2012, 2015, 2016; Cook et al., 2014; Cuaya et al, 2016; Dilks et al, 2015; Huber et al 2017; Horowitz, 2014; Jia et al., 2014, 2016; Kyathanahally et al, 2015; Thompkins et al, 2016; Ramahihgari et al, 2018). The selected "target" regions were: anterior cingulate cortex (ACC), ventromedial prefrontal cortex (vmPFC), bilateral dorsolateral prefrontal cortex (dlPFC), bilateral ventrolateral prefrontal cortex (vlPFC), posterior cingulate cortex (PCC), bilateral inferior parietal lobule (IPL), visual cortex, bilateral caudate, bilateral amygdala, olfactory bulb, bilateral hippocampus and bilateral temporal cortex. The MNI coordinates for these "target" regions in humans are listed in Table S2. For the dogs, these regions were manually marked (in our own custom standardized space as discussed in our previous publications (Jia et al., 2014, 2016; Kyathanahally et al., 2015; Thompkins et al., 2016)) by authors who were well versed with canine neuroanatomy. It is noteworthy that we have used a nomenclature involving the position of the regions in different cortices (using words such as bilateral, dorsal, posterior, inferior etc) and have avoided referring to specific gyri and sulci which have different nomenclatures in humans and dogs.

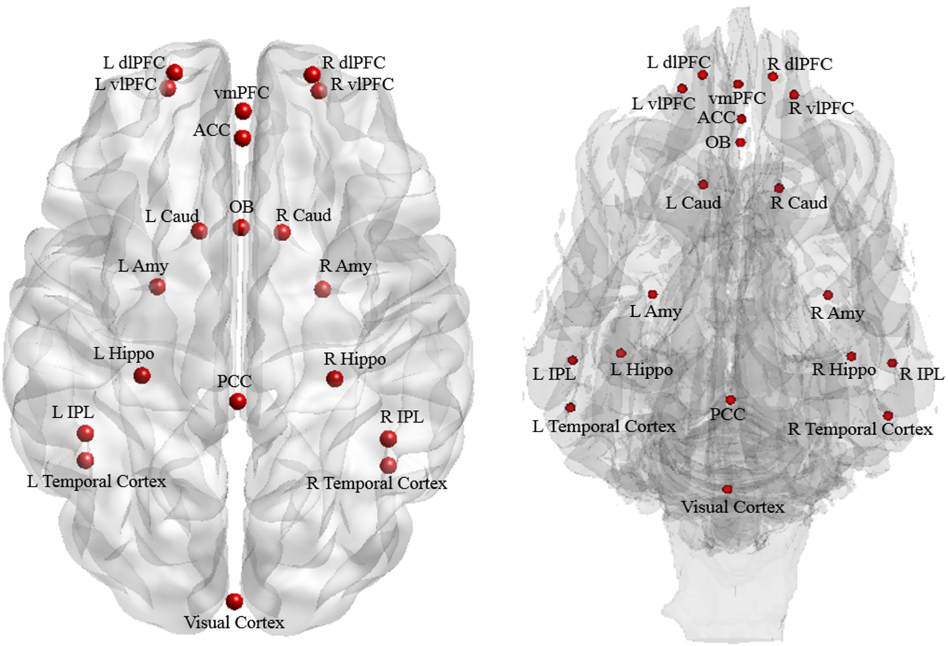

(a) (b)

Figure S1. A pictorial spatial representation of "target" regions in human brain (a) and dog brain (b). ROI abbreviations: anterior cingulate cortex (ACC), ventromedial prefrontal cortex (vmPFC), bilateral dorsolateral prefrontal cortex (dlPFC), bilateral ventrolateral prefrontal cortex (vlPFC), posterior cingulate cortex (PCC), bilateral inferior parietal lobule (IPL), bilateral Hippocampus (Hippo), bilateral amygdala (Amy), bilateral caudate (Caud), olfactory bulb (OB)

Table S2. Montreal Neurological Institute (MNI) coordinates of "target" regions in humans. ROI abbreviations as in Fig. S1

	**MNI coordinates (x, y, z)**		
	**X**	**Y**	**Z**
ACC	0	40	8
vmPFC	0	50	-7
dlPFC	+/-20	56	28
vlPFC	+/-22	57	-12
PCC	0	-40	27
IPL	+/-44	-52	40
Visual Cortex	0	-94	-6
Caudate	+/-12	12	12
Amygdala	+/-24	0	-20
Olfactory bulb	0	16	-8

Hippocampus	+/-28	-28	-8
Temporal cortex	+/-44	-52	-16

Seed regions in the dog brain were defined as regions involving paths whose changes in connectivity with time correlated with corresponding behavioral changes due to detection training (more on this in the results section). The seed regions in the human brain included every voxel in the human brain. The connectivity fingerprints of "seed" regions for each subject were determined based on the correlation of time series of seed regions (mean time series of seed ROIs in dogs and just the voxel time series in humans) with those of "target" regions. It is noteworthy that the number of "targets" should be sufficient enough to capture the diversity of the connectivity from the seed regions, but should not be too many that might cause overfitting.

Then the Manhattan distance between the averaged connectivity fingerprint of each dog seed and each human seed (averaged over the dog and human samples, respectively) were calculated to determine voxels in the human brain that share the same pattern or fingerprint of connectivity with "seed" regions in dogs (Fig. S2). If the connectivity fingerprints are represented as vectors x and y of dimension n, then the Manhattan distance between them is defined as $\sum_{i=1}^{n}|x_i - y_i|$. Permutation testing was used to test the significance of the match between each of voxels in the human brain and the dog "seed" regions ($p < 0.01$). This established a statistical criterion to regions in the human brain which were functionally analogous to the selected region in the dog brain. Even though this method does not impose an anatomical constraint on the location of the identified homologous region, results from previous studies (Mars et al., 2016; Passingham et al., 2002) as well as ours (presented in the results section) suggest that the identified homolous regions made sense from an anatomical perspective.

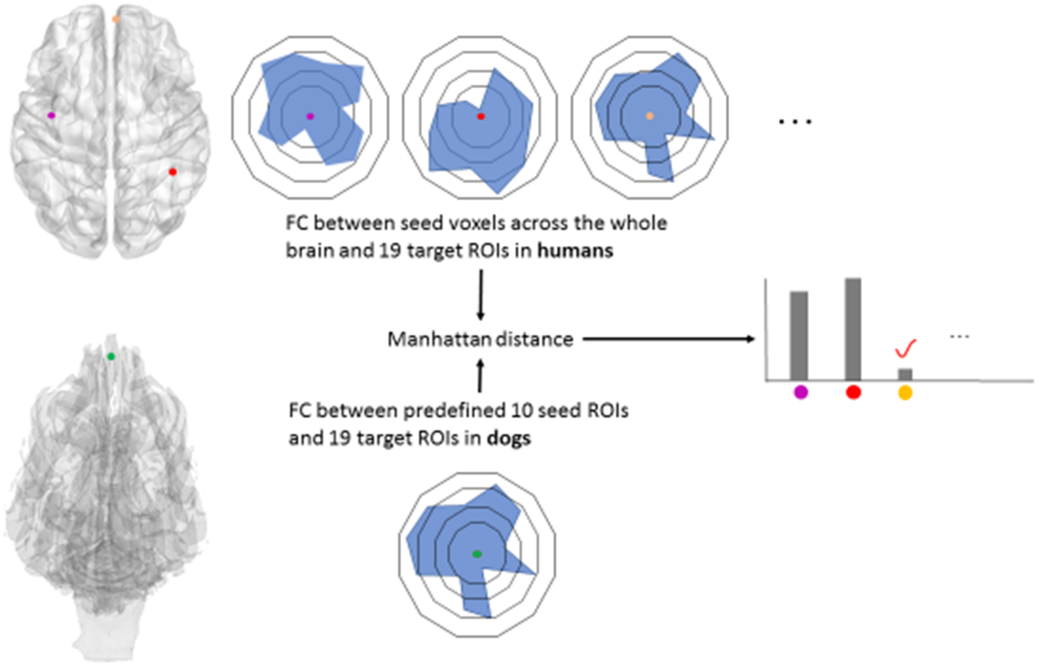

Figure S2. A schematic illustration of the connectivity fingerprint matching approach in our study. Each vertex of the polygon indicates individual "target" regions. A connectivity fingerprint of a dog "seed" region (in *green*) is compared to connectivity fingerprints obtained from human "seed" voxels picked from across the entire human brain (here we randomly selected three voxels shown in *yellow*, *purple* and *red* for illustration). The connectivity fingerprints were calculated by estimating resting state functional connectivity between the seed regions and 19 pre-selected target regions. Manhattan distance measure was used to determine the similarity of different fingerprints. In this case, the yellow area in the human brain has a connectivity fingerprint with target regions that are most similar to that obtained by the dog "seed" region. Therefore, the yellow voxel in the human brain could potentially be the region homologous to the green seed voxel in the dog brain.

RESULTS

To better understand the functional roles of regions in the dog brain connected by paths identified above, corresponding homologous regions in the human brain were statistically determined using the permutation test ($p<0.05$). This was done because there is a lot more literature about the functional roles of brain regions in the human brain than in the dog brain. We selected 15 "seed" regions from the dog brain based on regions that were connected in the paths identified above (R Pyri, R IPL, L Pyri, L IPL, L Claustrum/Insula, L Hippo, L Amy, Hypo, Brainstem, L MFG, R Caud, R Claustrum/Insula, OB, R DLPFC, and R IFG). The role of many of these regions in canine cognition is unclear due to lack of corresponding literature. After identifying the homologous regions in the human brain (Table S3), corresponding paths connecting them

in the human brain were mapped on a brain surface (Fig. S3) using BrainNet Viewer software (Xia, Wang, & He, 2013).

Table S3. Regions of the dog brain connected by paths identified above (in Table 1) and corresponding homologous regions in the human brain with their MNI coordinates. Note that L Hippo, L Amy, Hypo, R Caud and OB in the dog brain mapped onto regions in the human brain with exactly the same nomenclature and hence they are not shown in the Table below.

Dog region	Human region	Peak MNI coordinate in human brain		
		X	y	Z
R pyriform lobe	R parahippocampal gyrus	14	-8	-22
R inferior parietal lobe	R inferior parietal lobe	36	-46	39
L pyriform	L parahippocampal gyrus	-16	-4	-14
L inferior parietal lobe	L inferior parietal lobe	-38	-44	50
L Claustrum/Insula	L Claustrum/Insula	-38	6	4
Brainstem	Locus Coeruleus in Brainstem	2	-40	-36
L middle frontal gyrus	L middle frontal gyrus	-40	48	14
R Claustrum/Insula	R Insula	40	6	2
R dorsolateral prefrontal cortex	R superior frontal gyrus	16	46	30
R inferior frontal gyrus	R inferior frontal gyrus	60	10	22

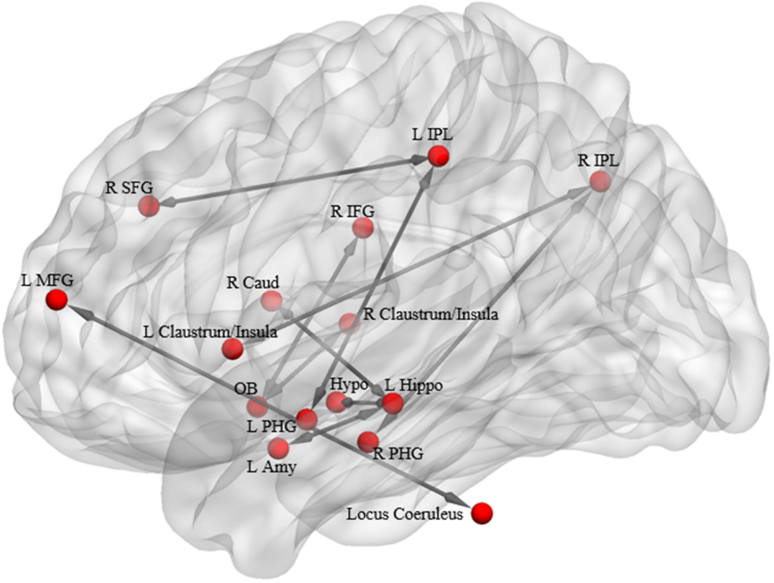

Figure S3. A pictorial spatial representation of homologous regions in the human brain identified in Table. S3. *PHG = parahippocampal gyrus, IPL = inferior parietal lobe, Hippo = hippocampus, Amy = amygdala,*

Hypo = hypothalamus, MFG = middle frontal gyrus, Caud = caudate, OB = olfactory bulb, SFG = superior frontal gyrus, IFG = inferior frontal gyrus

The corresponding homologous regions (Table S4) in the human brain and corresponding paths were then mapped on a human brain surface (Fig. S4). The success of the predictive model was not influenced by dropouts since only dogs which had usable data at all time points were used in the analysis.

Table S4. Regions of the dog brain connected by paths identified above (in Table 3) and corresponding homologous regions in the human brain with their MNI coordinates

Dog region	Human region	Peak MNI coordinate in human brain		
		x	y	z
L caudate	L caudate	-12	2	20
L middle temporal gyrus	L middle temporal gyrus	-58	-46	6
R superior temporal gyrus	R superior temporal gyrus	54	-40	12

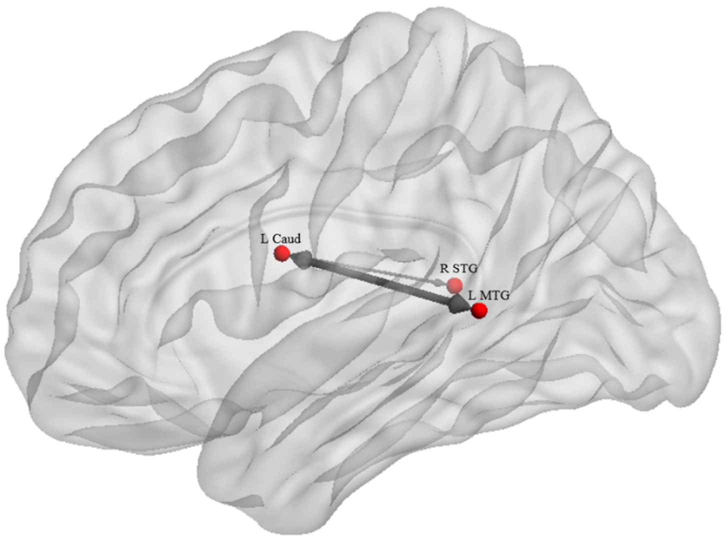

Figure S4. A pictorial spatial representation of homologous regions in the human brain identified in Table. S4. The thick line corresponds to multiple paths between L Caud and L MTG while the thin line corresponds to the single connection between L Caud and R STG. *Caud = Caudate, MTG = middle temporal gyrus, STG = superior temporal gyrus*
